# Supplementary material for: TEAD Inhibitors Sensitize KRASG12C Inhibitors via Dual Cell Cycle Arrest in KRASG12C-Mutant NSCLC
Source: Pharmaceuticals (Basel). 2023 Apr 6;16(4):553. doi: 10.3390/ph16040553 (PMC10142471; doi:10.3390/ph16040553)
Supplement: Supplementary file 1 [file pharmaceuticals-16-00553-s001.zip › pharmaceuticals-2266435-supplementary.pdf]

Supplementary information

Supplementary Figure S1

A

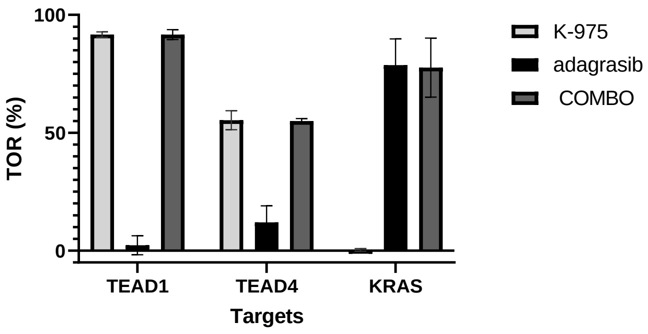

B

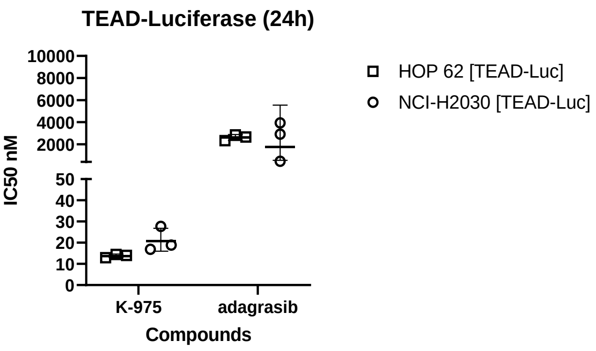

C

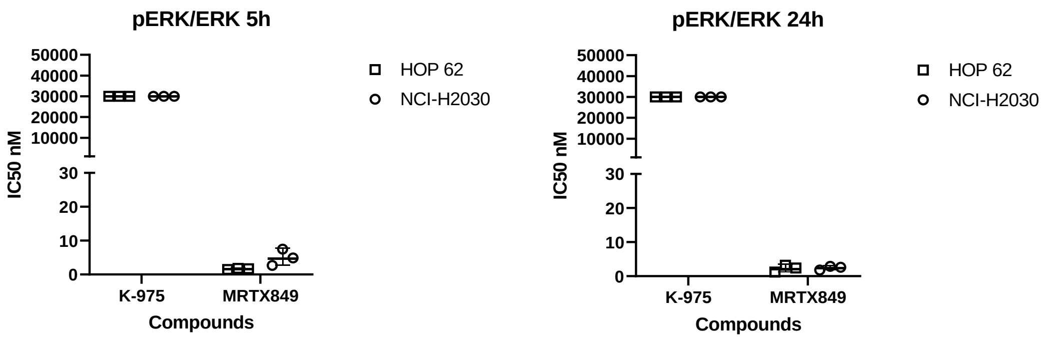

Supplementary Figure S1. *Validation of the engagement of TEAD and $KRAS^{G12C}$ targets.* (A) TOR observed for TEAD1, TEAD4 and $KRAS^{G12C}$ in the NCI-H2030 cell line, for the conditions tested. **(B)** TEAD-Luciferase activity monitored in cell lines modified by lentivirus HOP-62[TEAD-Luc] and NCI-H2030[TEAD-Luc] at 24 h for the K-975 and adagrasib treatment. **(C)** Modulation of phosphorylation of ERK1/2 proteins at the Thr202/Tyr204 and Thr185/Tyr187 sites at 5 h and 24 h in KRASG12C mutated cell lines HOP 62 and NCI-H2030 for the K-975 and adagrasib treatment.

Supplementary Figure S2

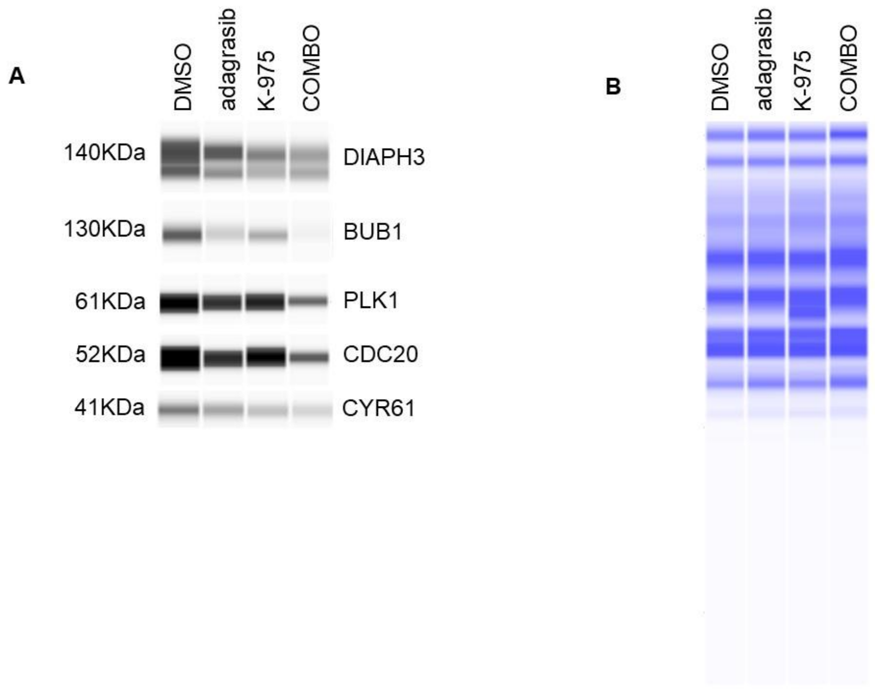

Supplementary Figure S2. *Validation of the biomarker modulation by KRAS^{G12C}/TEAD combination.* (A) Representative figure of Western blot from the Jess technology. Conditions tested: DMSO, adagrasib, K-975 and COMBO. We monitored the modulation at 24h looking at some biomarkers identified. The data set is on 3 biological replicates. (B) Representative figure of Total Protein from Jess technology (WB via capillarity).

Supplementary Figure S3

A

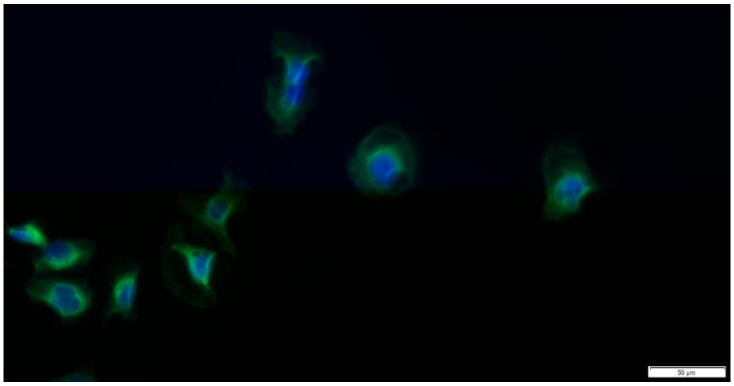

Control – DMSO

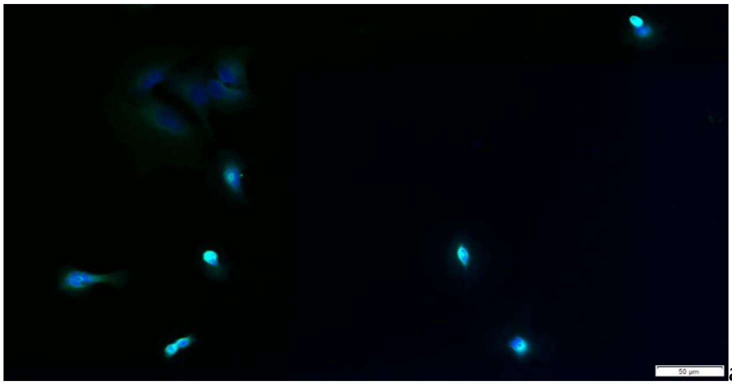

adagrasib

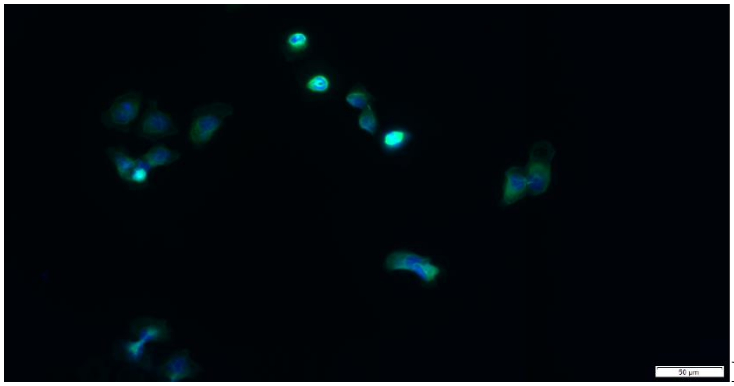

K-975

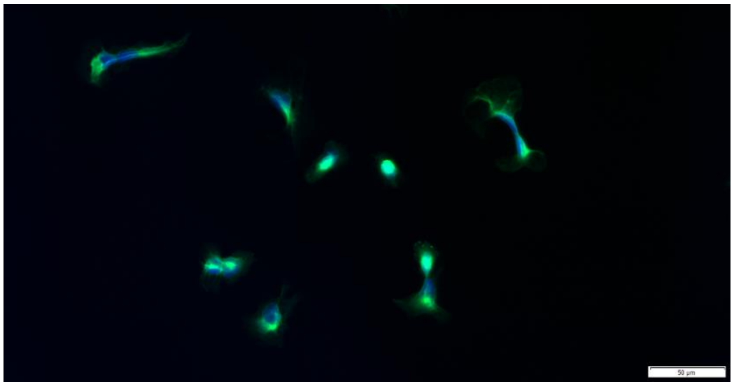

COMBO

B

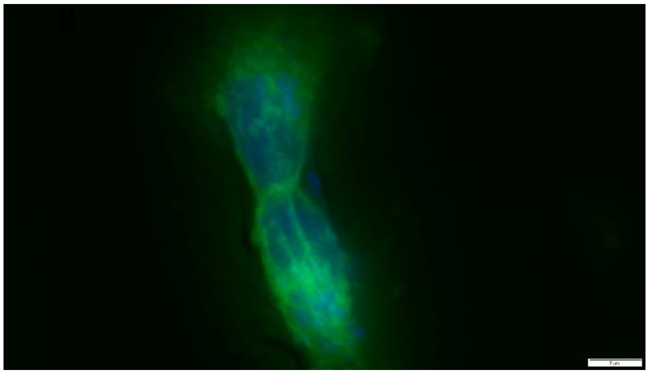

Control – DMSO

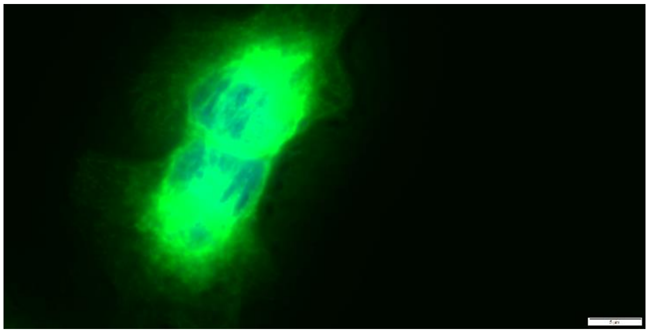

COMBO

Supplementary Figure S3. *Tubulin condensation is increased by $KRAS^{G12C}$i/TEADi combination.* (A) Tubulin density was detected at 24h. Beta-tubulin (green) and DNA (blue) detection via IHC in the 4 conditions tested: DMSO, adagrasib (30 nM), K-975 (100 nM) and COMBO (adagrasib and K-975 at the respective doses). Images at 50 µm. (B) Representative figure to see tubulin increase density in COMBO compared to DMSO. Image at 5 µm.

Supplementary Figure S4

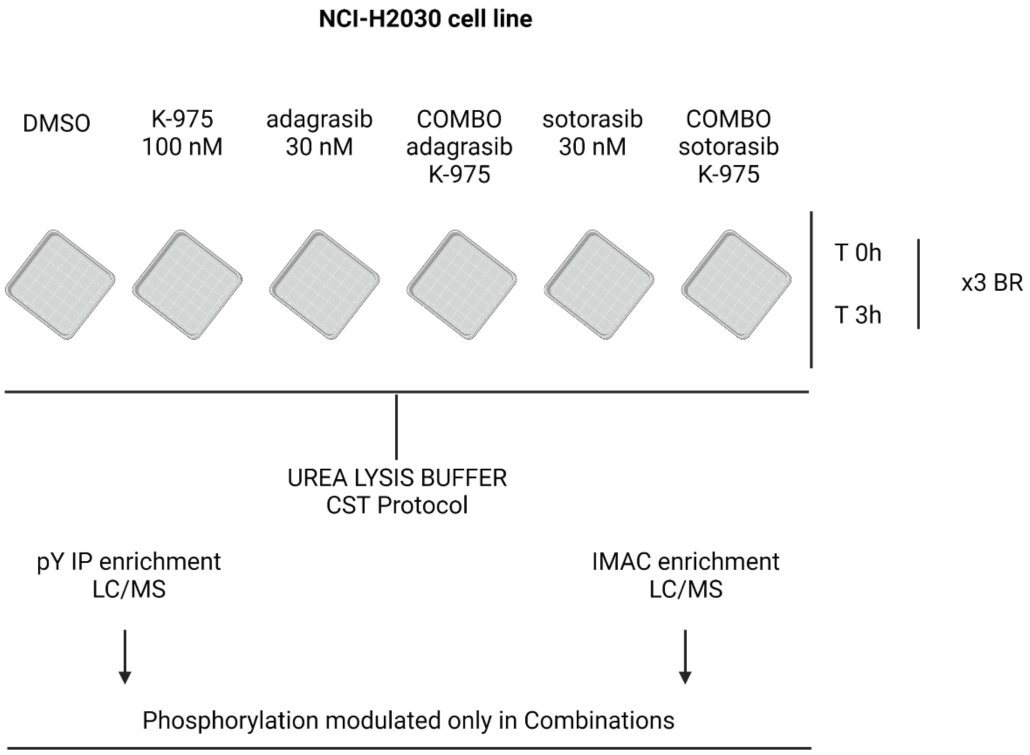

Supplementary Figure S4. PTMScan procedure. *Description of the PTMScan procedure following the instruction of CST*. NCI-H2030 cell lines were seeded in 300 cm^2 dishes. After 72 h, time 0 h (T 0 h) was collected and the rest was treated with single agents or in combination as shown. At time 3 h (T 3 h) cells were collected and lysate in Urea Lysis Buffer (following the CST protocol). Cell lysates were sent to CST to be used for enrichment via immunoprecipitation with phosphotyrosine (pY) antibody followed by mass spectrometry or Immobilized Metal Affinity Chromatography (IMAC) followed by mass spectrometry. The phosphopeptites of interest were selected.

Supplementary Figure S5

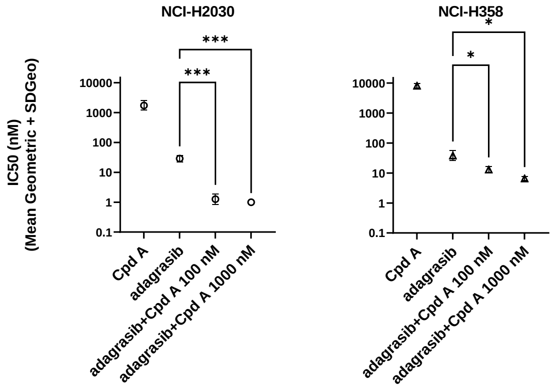

Supplementary Figure S5. *TEADi cpd A increases the response to KRAS^{G12C}i adagrasib in NCI-H2030 and NCI-H358 cell lines*. IC_{50} detection at 144 h after treatment for the single and the combination treatments with adagrasib and K-975. Geometric mean and SD were calculated from a minimum of three biological replicates for each cell line.

Supplementary Table S1.Targets Venn Diagram

Specific effect Combination-Direct targets (TEAD responsive elements present)		
**Gene name**	**Gene desciotion**	**Classification based on Bibliography studies**
ATAD2	ATPase Family AAA Domain Containing 2	S phase/DNA Synthesis or Regulation
RRM2	Ribonucleotide Reductase Regulatory Subunit M2	S phase/DNA Synthesis or Regulation
PRIM2	DNA Primase Subunit 2	S phase/DNA Synthesis or Regulation
UHRF1	Ubiquitin Like With PHD And Ring Finger Domains 1	S phase/DNA Synthesis or Regulation
ECT2	Epithelial Cell Transforming 2	G2M-Mitosis-Mitotic spindle Regulation or Cytokinesis
DIAPH3	Diaphanous Related Formin 3	G2M-Mitosis-Mitotic spindle Regulation or Cytokinesis
SMTN	Smoothelin	Cytoskeleton Cell-cell interation
CDC6	Cell Division Cycle 6	Cell cycle regulation
CCNA2	Cyclin A2	Cell cycle regulation
ODC1	Ornithine Decarboxylase 1	Other functions or processes
EPHA2	EPH Receptor A2	Other functions or processes
SLC4A7	Solute Carrier Family 4 Member 7	Other functions or processes
PDCD4	Programmed Cell Death 4	Other functions or processes
RHOB	Ras Homolog Family Member B	Other functions or processes
**Specific effect Combination-Indirect targets(TEAD responsive elements not present)**		
**Gene name**	**Gene desciotion**	**Bibliography studies**
HAUS6	Augmin Like Complex Subunit 6	Mitotic spindle
FAM83D	Family with Sequence Similarity 83 Member D	Mitotic spindle
MCM10	Minichromosome Maintenance 10 Replication Initiation Factor	Modulation in Proteomic was below the detection level in all condition
CDC20	Cell Division Cycle 20	G2M-Mitosis-Mitotic spindle Regulation or Cytokinesis
HELLS	Helicase, Lymphoid Specific	S phase/DNA Synthesis or Regulation
TIPIN	TIMELESS Interacting Protein	S phase/DNA Synthesis or Regulation
HMMR	Hyaluronan Mediated Motility Receptor	Mitotic spindle
CDCA3	Cell Division Cycle Associated 3	G2M-Mitosis-Mitotic spindle Regulation or Cytokinesis
PLK1	Polo Like Kinase 1	G2M-Mitosis-Mitotic spindle Regulation or Cytokinesis
UBE2S	Ubiquitin Conjugating Enzyme E2 S/C	G2M-Mitosis-Mitotic spindle Regulation or Cytokinesis
GTSE1	G2 And S-Phase Expressed 1	G2M-Mitosis-Mitotic spindle Regulation or Cytokinesis
SHCBP1	SHC Binding And Spindle Associated 1	Mitotic spindle
UBE2C	Ubiquitin Conjugating Enzyme E2 S/C	G2M-Mitosis-Mitotic spindle Regulation or Cytokinesis
KIFC1	Kinesin Family Member C1	G2M-Mitosis-Mitotic spindle Regulation or Cytokinesis
AURKB	Aurora B kinase	G2M-Mitosis-Mitotic spindle Regulation or Cytokinesis
UBE2T	Ubiquitin Conjugating Enzyme E2 T	Other functions or processes
PBXIP1	PBX Homeobox Interacting Protein 1	Other functions or processes
THBS3	Thrombospondin 3	Other functions or processes
EPB41L1	Erythrocyte Membrane Protein Band 4.1 Like 1	Other functions or processes

Supplementary Table S1. *Target Venn Diagram*. Listed genes that are modulated specifically by KRAS and YAP1-TEAD presenting TEAD responsive elements (direct target) and non-presenting TEAD responsive elements (indirect target).

Supplementary Table S2. pY and IMAC phosphosites identified

Supplementary Table S2. pY and IMAC phosphodites identified.

pY 3h

Gene.Name	Description	sotorasib	adagrasib	K-975	sotorabis+K-975	adagrasib+K-975	Site (pTyr)
TUBB	Tubulin beta chain	0,16	0,05	-0,64	-2,39	-2,29	§50
PKP4	Plakophilin-4	0,09	-0,82	-0,37	-1,24	-1,78	§1051
ZNF528;ZNF528	Zinc finger protein 528	-0,26	-0,75	0,12	-1,42	-1,3	537;304
LARP6	La-related protein 6	0,73	0,95	-0,2	-1,11	-1,18	126
OSMR	Oncostatin-M-specific receptor subunit beta	-0,55	-0,74	-0,07	-1,1	-1,14	§861
GSTP1;LGALS8;LGALS8	Glutathione S-transferase P	-0,62	-0,78	-0,95	-1,04	-1,13	§80;§141;141
CDK16;CDK16;CDK16;CDK17;CDK17	Cyclin-dependent kinase 16	0,52	-0,57	0,84	2	1,01	§178;252;184;§205;205

IMAC 3h

Gene.Name	Description	sotorasib	adagrasib	K-975	sotorabis+K-975	adagrasib+K-975	Site (pThr or pSer)
UBAP2L;UBAP2L;UBAP2L;UBAP2L;UBAP2L	Ubiquitin-associated protein 2-like	-0,95	-0,08	0,29	-3,15	-2,25	§470, §477;§470, §477;§470, §477;463, §470;470, 477
STIM2;STIM2	Stromal interaction molecule 2	-0,78	-0,96	-0,37	-1,16	-1,85	§697;792
CAMSAP2;CAMSAP2;CAMSAP2	Calmodulin-regulated spectrin-associated protein 2	-0,49	-0,88	-0,58	-1,12	-1,26	§1268;1241;1257
SLC4A4;SLC4A4;SLC4A4;SLC4A4;SLC4A4	Electrogenic sodium bicarbonate cotransporter 1	-0,25	0	-0,01	-1	-1,18	§254;210;210;§254;254
ARFGEF2	Brefeldin A-inhibited guanine nucleotide-exchange protein 2	-0,83	-0,27	-0,73	-1,06	-1,07	§1528
CACTIN;CACTIN	Cactin	0,23	0,64	-0,1	1,3	1,01	§71, §73;71, 73
PHLDB2;PHLDB2;PHLDB2	Pleckstrin homology-like domain family B member 2	0,24	0,12	0,41	1,04	1,15	§308;308;335
CALM1;CALM2;CALM3	Calmodulin-1	0,22	0,52	0,12	1,02	1,2	§82;§82;§82
CUL4B;CUL4B	Cullin-4B	0,51	0,58	0,49	1,02	1,24	§85;§67
RACGAP1	Rac GTPase-activating protein 1	0,67	0,64	0,58	1,08	1,28	§342
WIPF2;WIPF2	WAS/WASL-interacting protein family member 2	0,88	0,86	0,7	1,09	1,35	300;150
WIPF2;WIPF2	WAS/WASL-interacting protein family member 2	0,88	0,86	0,7	1,09	1,35	303;153
TRIM3;TRIM3;TRIM3;TRIM3	Tripartite motif-containing protein 3	0,66	0,55	-0,39	1,43	1,42	§455, §458;444, 447;376, 379;336, 339
TRIM3;TRIM3;TRIM3;TRIM3	Tripartite motif-containing protein 3	0,66	0,55	-0,39	1,43	1,42	§457, §458;446, 447;378, 379;338, 339
KMT2D;KMT2D	Histone-lysine N-methyltransferase 2D	0,32	0,07	0,76	1,02	1,45	§2240;2243
KMT2D;KMT2D	Histone-lysine N-methyltransferase 2D	0,53	-0,1	-0,41	1,22	1,5	§1843;1846
IK	Protein Red	-0,09	-0,17	-0,07	1,07	1,56	§42
SIPA1L2	Signal-induced proliferation-associated 1-like protein 2	-0,07	-0,16	-0,51	1,08	1,58	§194
EXOSC1	Exosome complex component CSL4	0,49	0,35	0,57	1,05	1,63	§98
DBF4	Protein DBF4 homolog A	0,71	-0,77	-0,32	1,17	1,73	§345
BCLAF3;BCLAF3;BCLAF3	BCLAF1 and THRAP3 family member 3	0,26	-0,43	0,69	1,58	1,79	§402;402;402
FASN	Fatty acid synthase	0,34	0,04	0,49	1,19	1,83	724
ZHX2	Zinc fingers and homeoboxes protein 2	0,42	-0,41	-0,04	1,41	2	§37
RANBP2;RGPD1;RGPD2;RGPD3;RGPD4;RGPD5;RGPD[illegible]	E3 SUMO-protein ligase RanBP2	0,01	-0,96	-0,15	1,33	2,07	§2153;§1162;§1170;§1178;§1178;§1177;§1177
ETV6	Transcription factor ETV6	0,27	-0,3	0,5	1,3	2,95	§271

Supplementary Table S2. *pY and IMAC phosphosites identified***.** Genes specifically modulated after 3 h treatment of combination treatment (sotorasib with K-975 or adagrasib with K-975). Downregulated genes shown in blue and upregulated in red. Values shown are normalized fold changes compared with DMSO.
